# Tumor-infiltrating T cells as a risk factor for lymph node metastasis in patients with submucosal colorectal cancer

**DOI:** 10.1038/s41598-023-29260-1

**Published:** 2023-02-06

**Authors:** Masatoshi Kitakaze, Shiki Fujino, Norikatsu Miyoshi, Yuki Sekido, Tsuyoshi Hata, Takayuki Ogino, Hidekazu Takahashi, Mamoru Uemura, Tsunekazu Mizushima, Yuichiro Doki, Hidetoshi Eguchi

**Affiliations:** 1 Department of Gastroenterological Surgery, Osaka University Graduate School of Medicine, 2-2 Yamadaoka, Suita, Osaka 565-0871 Japan; 2 Department of Gastroenterological Surgery, Minoh City Hospital, Minoh, Osaka 562-0014 Japan; 3 Department of Gastroenterological Surgery, Osaka Police Hospital, Osaka, Osaka 543-0035 Japan

**Keywords:** Colorectal cancer, Tumour immunology

## Abstract

Approximately 10% of patients with colorectal cancer with submucosal invasion have lymph node metastasis. Pathological risk factors for lymph node metastasis have varying sensitivities and specificities. To predict the risk of lymph node metastasis, the identification of new risk factors is vital. Tumor-infiltrating T cells have been reported to improve the prognosis of many solid tumors. Therefore, the purpose of this study was to examine the relationship between lymph node metastasis and tumor-infiltrating T cells in patients with colorectal cancer with submucosal invasion. We examined CD8^+^ tumor-infiltrating T cells level as a risk factor for lymph node metastasis in patients with colorectal cancer with submucosal invasion. Using immunohistochemical staining, we identified CD8 + T cells in surgically resected specimens from 98 patients with SM-CRC. We showed that low CD8^+^ tumor-infiltrating T cells levels are positively correlated with lymph node metastasis. Furthermore, by combining the number of CD8^+^ tumor-infiltrating T cell and the number of CD103^+^ tumor-infiltrating T cells, the results showed a high positive predictive value for lymph node metastasis in cases with low numbers of both types of tumor-infiltrating T cells and a high negative predictive value in cases with high numbers of both types of tumor-infiltrating T cells.

## Introduction

Approximately 10% of patients with colorectal cancer with submucosal invasion (SM-CRC) experience lymph node metastasis. For such patients, the Japanese Society for Cancer of the Colon and Rectum guidelines recommend surgical resection with lymph node dissection^1^.

For patients diagnosed with SM colorectal cancer after endoscopic treatment, additional surgical resection is performed based on pathological findings. The guidelines recommend additional resection in cases with one or more of the following pathologic factors related to lymph node metastasis: depth of submucosal invasion greater than 1000 μm, positive lymphovascular invasion, positive endoscopic vertical margin, poorly differentiated adenocarcinoma, signet-ring cell carcinoma, or mucinous carcinoma, and grade 2/3 budding at the site of the deepest invasion^1^. These pathological risk factors for lymph node metastasis have varying sensitivities and specificities. For example, a depth of submucosal invasion ≥ 1000 μm has a sensitivity of 94.8% and specificity of 19.0% for positive lymph node metastasis, while an undifferentiated histological type has a sensitivity of 13.3% and specificity of 97.9%^2^. The combination of these risk factors results in lymph node risks ranging from 7.4 to 46.9% in patients with SM-CRC^3^. Therefore, to predict the risk of lymph node metastasis, various methods, such as nomograms, have been utilized^4^.

The identification of new risk factors for lymph node metastasis in patients with SM-CRC is crucial^5,6^. In recent years, cancer immunity has been one of the most important factors associated with tumor prognosis^6^, and tumor-infiltrating T cells (TILs) have been reported to improve the prognosis of many solid tumors^7^. T cells can be classified as CD8^+^ or CD4^+^ T cells^8^. CD8^+^ T cells are cytotoxic T cells that contribute significantly to tumor prognosis^9^. Nevertheless, the role of CD8^+^ TILs in predicting lymph node metastasis in patients with SM-CRC has not been well evaluated. Although TILs in colorectal cancer have prognostic value^10^, the relationship between TILs and lymph node metastasis in patients with SM-CRC has not been reported.

Recently, CD103^+^ CD8^+^ T cells have been found to play an important role in cancer immunity, particularly in cancers of epithelial origin^11^.

The ligand for CD103 is the epithelial cell surface molecule E-cadherin, an epithelial-mesenchymal transition (EMT) marker^12^. Loss of E-cadherin in tumors is the hallmark of EMT and has been reported to be associated with poor prognosis for patients with colorectal cancer^13–15^. Therefore, we hypothesized that the number of infiltrating CD103^+^ cells is indirectly related to the EMT of the tumor and examined the relationship between the number of CD103^+^ TILs and lymph node metastasis.

Thus, the purpose of this study was to examine the relationship between lymph node metastasis and TILs in patients with SM-CRC.

## Results

### Patient’s characteristics and immunohistochemical staining of CD8 + TILs

**Figure 1:**
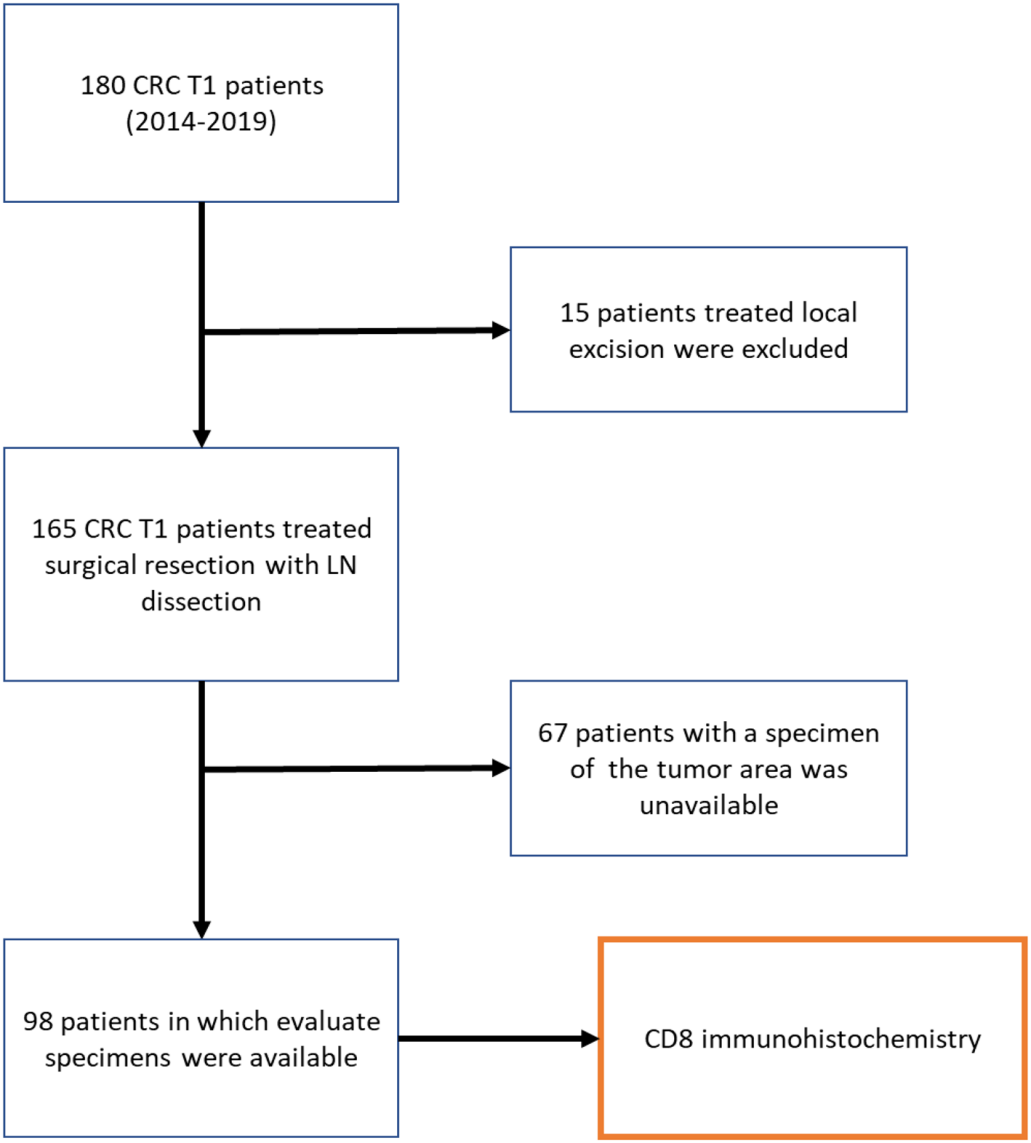
Scheme of the study. A total of 180 patients with surgically treated SM-CRC were enrolled in this study; 15 patients treated with local excision that lacked pathological lymph node metastasis data and 67 patients without available specimen of the tumor area were excluded.

We identified CD8^+^ T cells in surgically resected specimens from patients with SM-CRC using immunohistochemical staining with CD103 and CD8 antibodies. Of the total 180 eligible patients enrolled in this study, 15 patients treated with local excision that lacked lymph node dissection data and 67 patients without available tumor tissue were excluded. Finally, CD8^+^ TILs were evaluated in 98 cases (Fig. 1).

**Figure 2:**
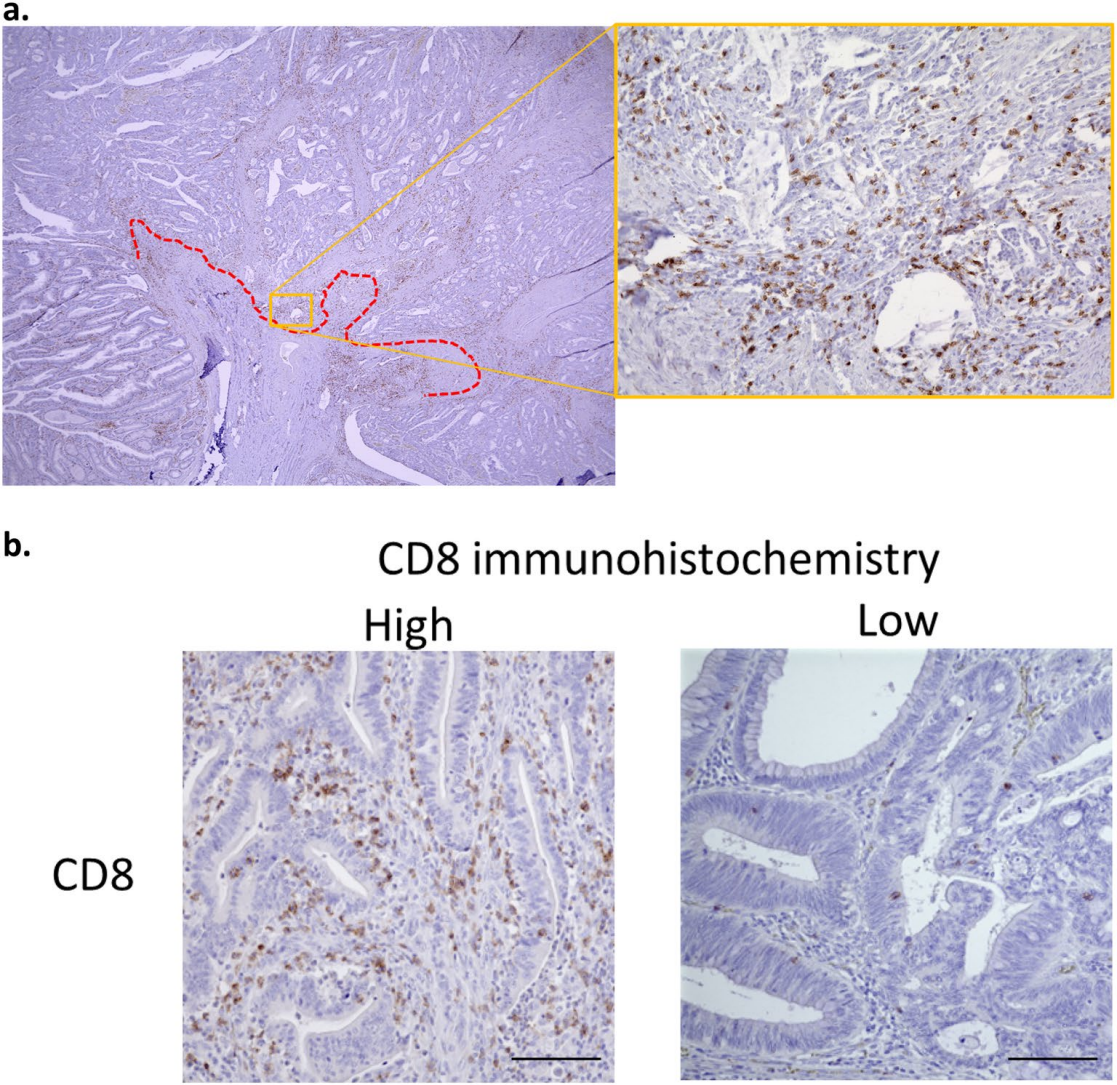
(**a**) The number of CD8^+^ TILs was counted at the invasive margin. The dotted line indicates the invasive margin. (**b**) Immunohistochemical staining for CD8 at the invasive margins of resected CRC tissue (representative images of a highly invasive tumor and low invasive tumor).

The evaluation site of the lymphocytes is shown in Fig. 2a. As shown in the figure, the invasive margin was set up, and the deepest part of the invasive margin with a large amount of tumor epithelial tissue was evaluated at × 200 magnification. Immunohistochemical staining showed variation among cases in the number of CD8^+^ TILs (Fig. 2b).

The characteristics of the 98 patients are presented in Table 1. Only two cases displayed undifferentiated histology, and lymph node metastasis was observed in 11 patients (11.2%). The median number of CD8^+^ TILs was 185 in 10 high-power fields (HPFs).

**Table 1:** Clinicopathologic factors of all patients.

Factor	N = 98
Age, median (years)	66 (35–86)
Sex [male/female]	58/40
Location [colon/rectum]	63/35
Histological type [tub1/tub2/por, muc]	53/43/2
Greatest diameter, median (mm)	20 (3–66)
Depth of invasion, median (μm)	2700 (100–35,000)
Lymphatic invasion [+ /-]	27/71
Vascular invasion [+ /-]	16/82
Budding grade [1/2/3/NA]	71/13/3/11
Preoperative CEA, median (ng/ml)	2 (0–21)
Preoperative CA19-9, median (ng/ml)	9 (0–243)
Number of CD8^+^ TILs (/10 HPF)	183 (10–1042)

*CEA* carcinoembryonic antigen, *CA19-9* Carbohydrate Antigen 19–9, *CD* cluster of differentiation, *TIL* tumor-infiltrating lymphocytes, *HPS* high-power fields.

### The number of CD8^+^ TILs and lymph node metastases

**Figure 3:**
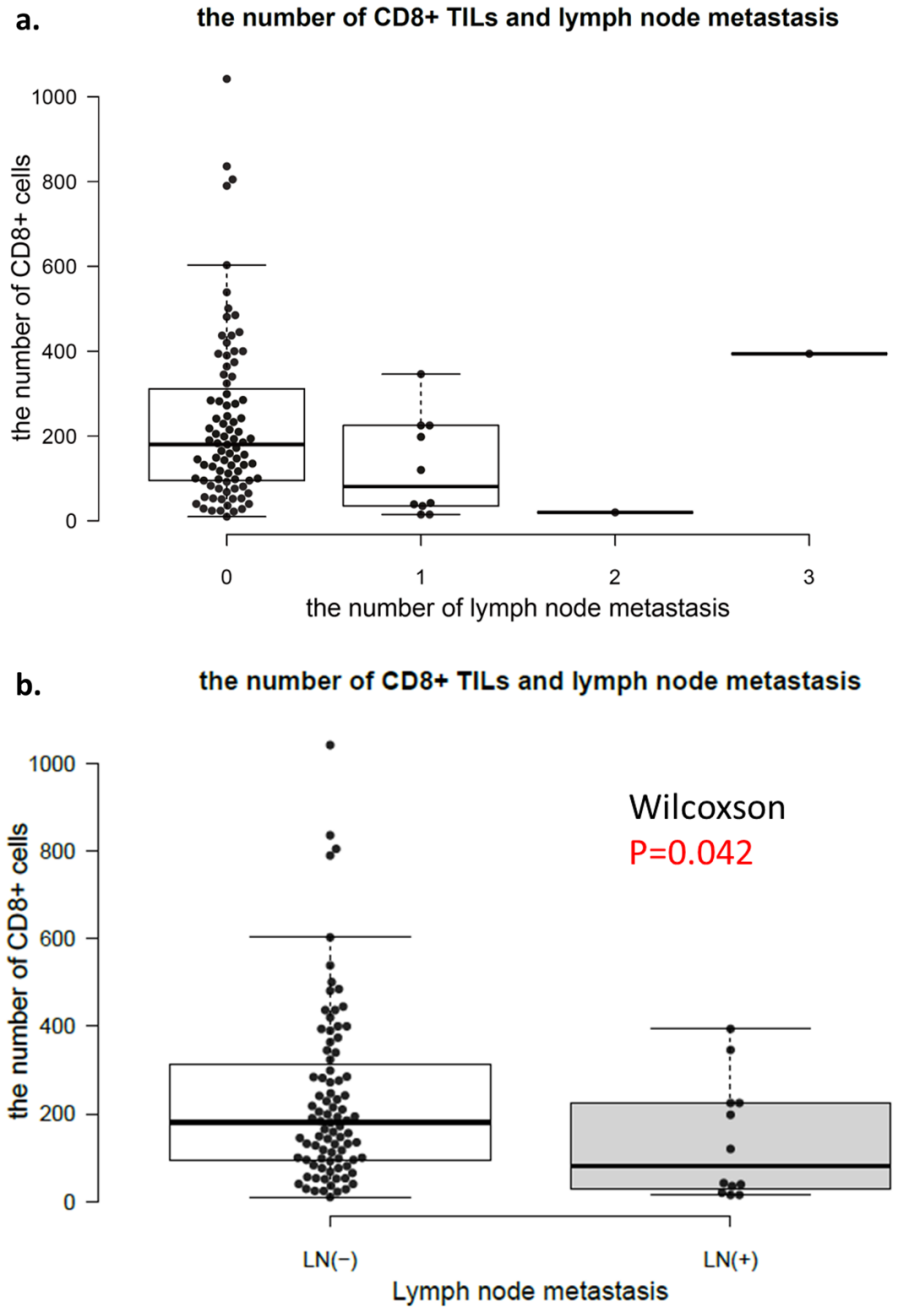
(**a**) The number of CD8^+^ TILs and lymph node metastases from SM-CRC. (**b**) The number of CD8^+^ TILs and presence of lymph node metastasis from SM-CRC (*P* = 0.042).

The number of CD8^+^ TILs decreased as the number of lymph node metastases increased (Fig. 3a), with the exception of one case with three lymph node metastases and a high number of CD8^+^ TILs.

The number of CD8^+^ TILs was significantly higher in cases without lymph node metastasis (median: 180/10 HPF, range 10–1042) than in those with lymph node metastasis (median: 42/10 HPF, range 15–394; *P* = 0.042; Fig. 3b).

**Figure 4:**
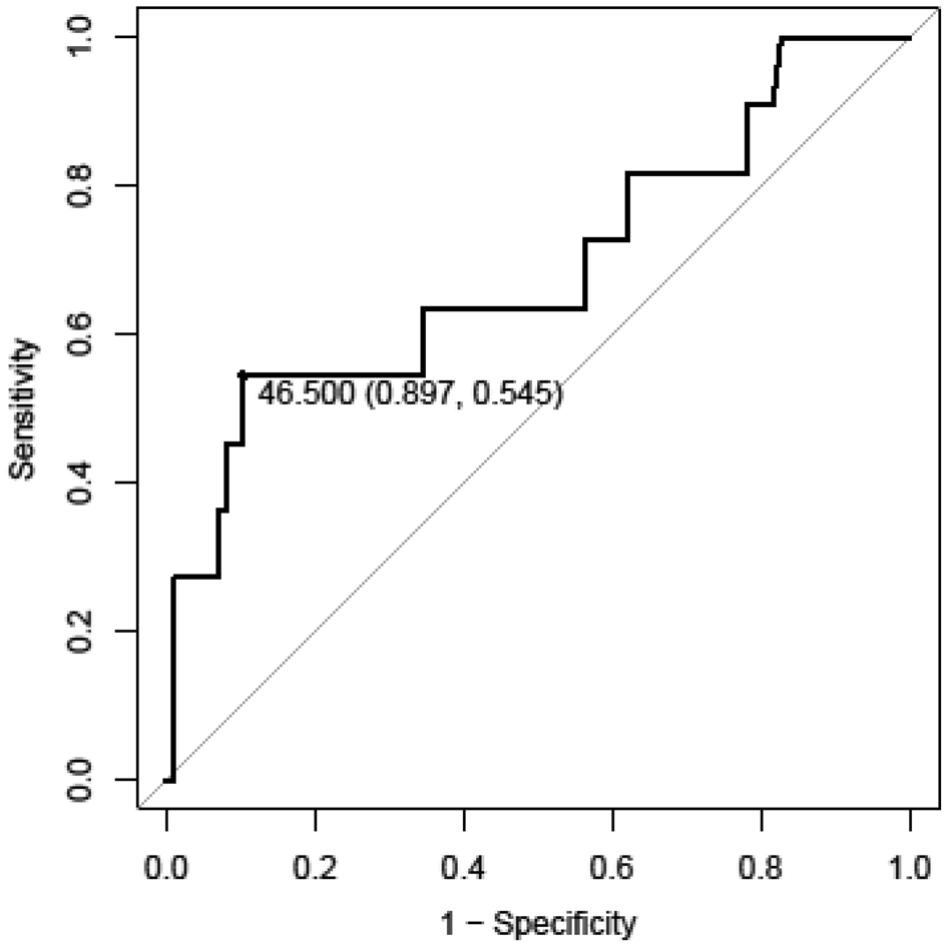
The receiver operating characteristic (ROC) curve of the relationship between the specificity and the sensitivity of the number of CD8^+^ TILs for lymph node metastasis.

The relationship between the specificity and sensitivity of the number of CD8^+^ TILs for the purpose of lymph node metastasis is represented by a receiver operating characteristic (ROC) curve (Fig. 4). The area under the ROC curve (AUC) was 0.689 for the number of CD8^+^ TILs.

The number of CD8^+^ TILs with the cutoff value identified by ROC curve analysis was compared with other clinicopathological risk factors for lymph node metastasis in univariate and multivariate analyses. Univariate analysis revealed that lymphatic invasion (+), budding grade (3), and CD8^+^ TILs (low) were risk factors for lymph node metastasis. Multivariate analysis confirmed that lymphatic invasion ( +) and the number of CD8^+^ TILs (low) were independent risk factors for lymph node metastasis.

The sensitivity, specificity, PPV, and NPV of each risk factor in the present dataset are shown in Table 2.

**Table 2:** Variables of lymph node metastasis (univariate and multivariate analyses).

Variable	Univariate			Multivariate		
	OR	95% CI	*P*-value	OR	95% CI	*P*-value
Age (≧ 65 years)	1.09	0.31–3.85	0.890			
Sex (female)	1.23	0.35–4.37	0.740			
Location (rectum)	1.03	0.28–3.80	0.962			
Histological type (por, muc)	–	–	–			
Histological type (tub2, por, muc)	1.48	0.41–5.21	0.544			
Greatest diameter (≧ 20 mm)	1.89	0.46–7.79	0.381			
Depth of invasion (≧ 1000 μm)	2.15	0.25–18.2	0.483			
Lymphatic invasion (+)	41.2	4.93–344	< 0.001	63.2	4.61–866	0.002
Vascular invasion (+)	1.16	0.23–5.95	0.860			
Budding grade (2, 3)	1.82	0.42–7.79	0.421			
Budding grade (3)	16.7	1.37–203	0.027	20.7	0.07–6523	0.302
Preoperative CEA (≧2 ng/ml)	2.75	0.55–13.8	0.219			
Preoperative CA19-9 (≧ 10 ng/ml)	1.54	0.40–5.87	0.529			
The number of CD8^+^ TILs (≦ 42/10 HPF)	10.4	2.64–41.0	< 0.001	16.9	1.46–195	0.024

*OR* odds ratio, *CI* confidence interval, *CEA* carcinoembryonic antigen, *CA19-9* Carbohydrate Antigen 19–9, *CD* cluster of differentiation, *TIL* tumor-infiltrating lymphocytes, *HPF* high-power fields.

### The combination of the number of CD8^+^ and CD103^+^ TILs and lymph node metastases

To identify a better indicator of lymph node metastasis, we evaluated the presence of CD103, another T cell activation marker.

**Figure 5:**
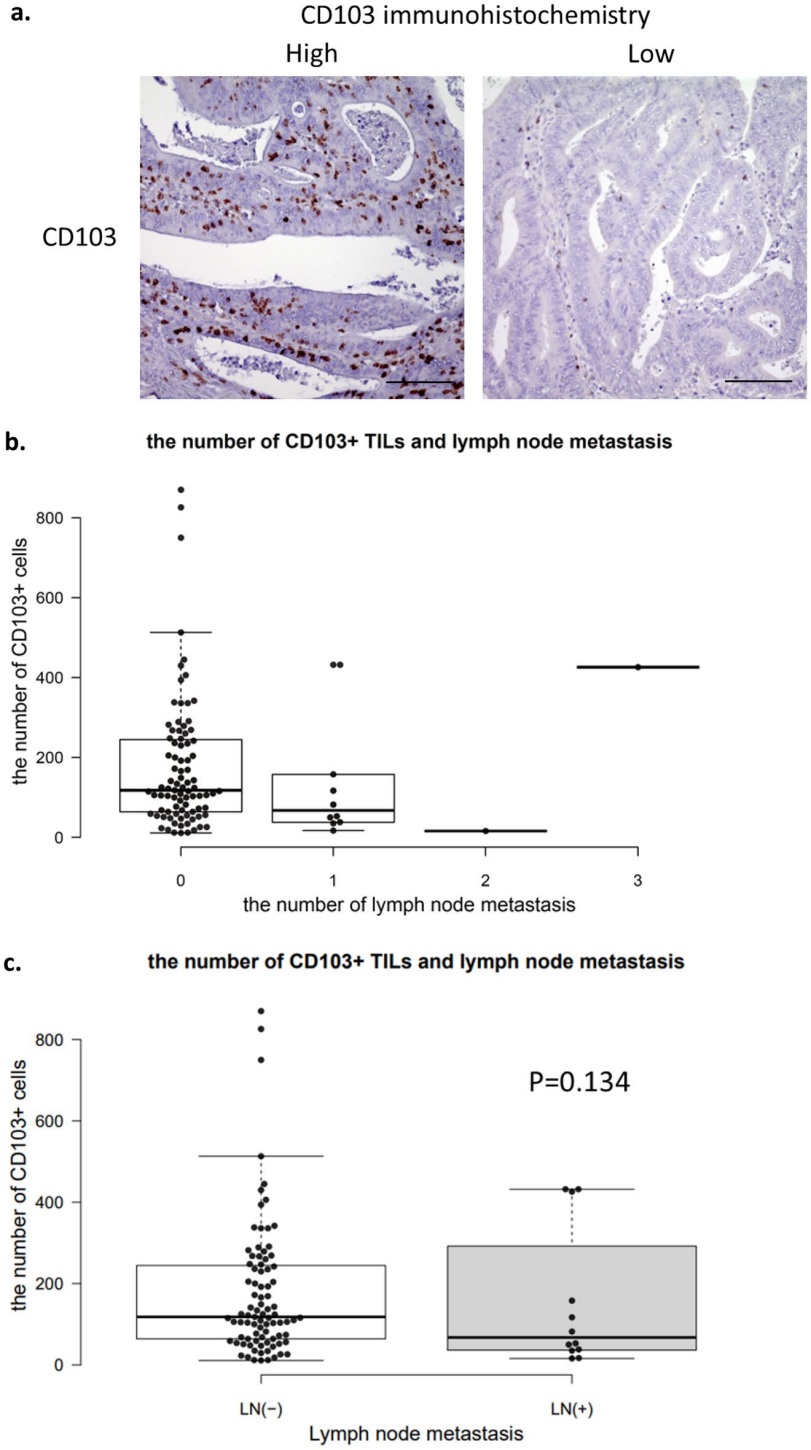
(**a**) Immunohistochemical staining for CD103 TILs at invasive margins of resected CRC tissue (left: high invasion; right: low invasion). (**b**) The number of CD103^+^ TILs and presence of lymph node metastasis from SM-CRC (*P* = 0.134). (**c**) The number of CD103^+^ TILs and lymph node metastases from SM-CRC.

Immunohistochemical staining showed that the number of CD103^+^ TILs varied among cases (Fig. 5a). The median number of CD103^+^ TILs was 116/10 HPFs. Furthermore, the number of CD103^+^ TILs was correlated with the number of CD8^+^ TILs (r = 0.524, *P* < 0.001).

**Figure 6:**
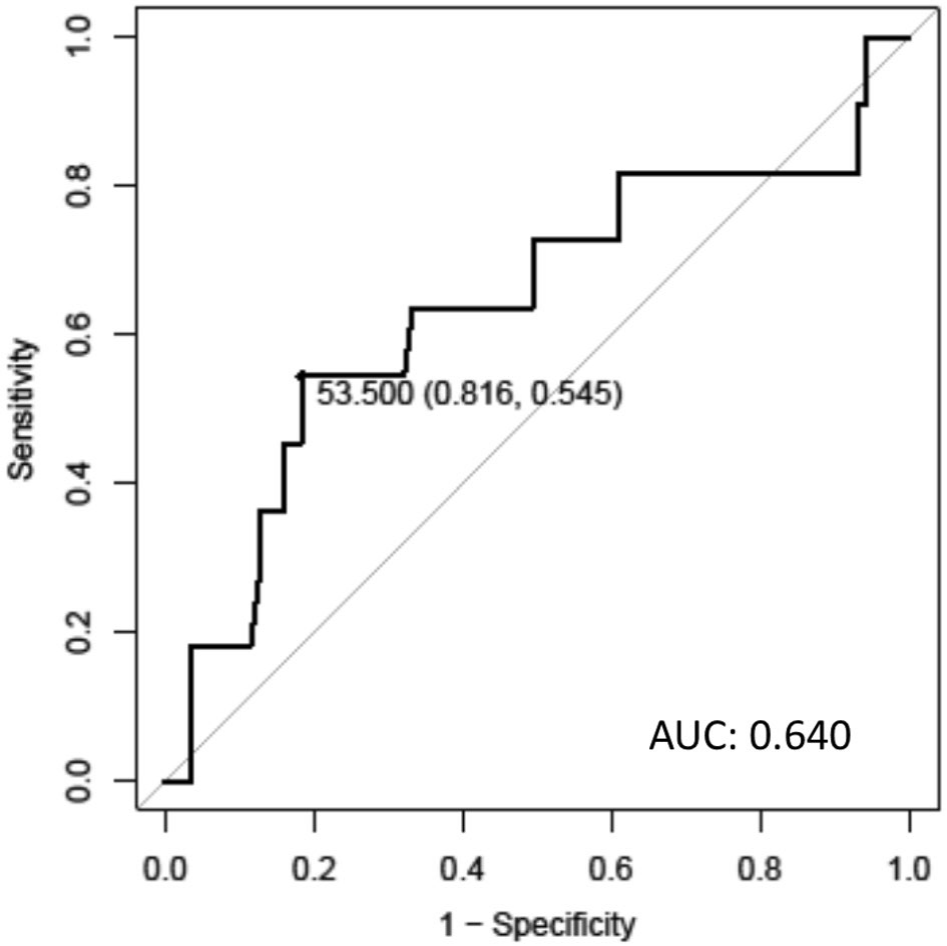
The receiver operating characteristic (ROC) curve of the relationship between the specificity and the sensitivity of the number of CD103^+^ TILs for lymph node metastasis.

The number of CD103^+^ TILs decreased as the number of lymph node metastases increased (Fig. 5b), except for one case with three lymph node metastases and a high number of CD103^+^ TILs. Moreover, the number of CD103^+^ TILs tended to be low in patients with lymph node metastasis; however, the difference was not statistically significant (*P* = 0.314; Fig. 5c). The AUC for the number of CD103^+^ TILs was 0.640 (Fig. 6).

The number of CD103^+^ TILs with the cutoff value identified by ROC curve analysis was compared with other clinicopathological risk factors for lymph node metastasis in univariate and multivariate analyses (Table 3). Univariate and multivariate analyses confirmed that CD103^+^ TILs (low) were an independent risk factor for lymph node metastasis.

**Table 3:** Factors evaluated for lymph node metastasis (univariate and multivariate analyses).

Lymph node metastasis	Univariate			Multivariate		
Variables	OR	95% CI	*P*-value	OR	95% CI	*P*-value
Age (≧ 65 year-old)	1.09	0.31–3.85	0.890			
Sex (Female)	1.23	0.35–4.37	0.740			
Location (Rectum)	1.03	0.28–3.80	0.962			
Histological type (por, muc)	–	–	–			
Histological type (tub2, por, muc)	1.48	0.41–5.21	0.544			
Greatest Diameter (≧ 20 mm)	1.89	0.46–7.79	0.381			
Depth of invasion (≧ 1000 μm)	2.15	0.25–18.2	0.483			
Lymphatic invasion (+)	41.2	4.93–344	< 0.001	50.1	4.97–504	< 0.001
Vascular invasion (+)	1.16	0.23–5.95	0.860			
Budding Grade (2, 3)	1.82	0.42–7.79	0.421			
Budding Grade (3)	16.7	1.37–203	0.027	11.6	0.05–2494	0.372
Preoperative CEA (≧ 2 ng/ml)	2.75	0.55–13.8	0.219			
Preoperative CA19-9 (≧ 10 ng/ml)	1.54	0.40–5.87	0.529			
The number of CD8^+^ TILs (< 46/10HPF)	10.4	2.64–41.0	< 0.001			
The number of CD103^+^ TILs (< 52/10HPF)	5.32	1.44–19.6	0.012	6.58	1.00–43.3	0.049

*OR* odds ratio, *CI* confidence interval, *CEA* carcinoembryonic antigen, *CA19-9* Carbohydrate Antigen 19–9, *CD* cluster of differentiation, *TIL* tumor-infiltrating lymphocytes, *HPS* high-power fields.

Clinicopathological risk factors for lymph node metastasis and the number of CD8^+^ TILs, CD103^+^ TILs, and CD8^+^ CD103^+^ TILs were compared using sensitivity, specificity, PPV, and NPV.

The NPVs of CD8^+^ TILs and CD103^+^ TILs were higher than those of other risk factors, except for lymphatic invasion (Table 4). The PPV of CD8^+^ CD103^+^ TILs was also higher than that of other risk factors (Table 4).

**Table 4:** Sensitivity, specificity, positive predictive value (PPV), and negative predictive value (NPV) of risk factors.

	Sensitivity	Specificity	PPV	NPV
Histological type (por, muc)	0	97.7	0	88.5
Histological type (tub2, por, muc)	54.5	55.2	13.3	90.6
Depth of invasion (≧ 1000 μm)	90.9	16.1	12.7	93.3
Lymphatic invasion (+)	90.9	80.5	37	98.6
Vascular invasion (+)	18.2	83.9	12.5	89
Budding grade (2, 3)	27.3	72.4	18.8	88.7
Budding grade (3)	18.2	86.2	66.7	89.3
CD8	54.5	89.7	40	94
CD103	54.5	81.6	27.3	93.4
CD8 and CD103 (LL/HH)	45.5	97.7	71.4	93.4
CD8 and CD103 (Other/HH)	63.6	73.6	23.3	94.1

*CD* cluster of differentiation, *LL* numbers of both CD103^+^ and CD8^+^ TILs were low, *HH* numbers of both CD8^+^ and CD103^+^ TILs were high.

We added some examinations under different conditions. First, we decided to examine pT1b cases, which are considered more likely to have lymph node metastasis. 78 pT1b cases were included, and 10 cases were associated with lymph node metastasis. The relationship between CD8^+^ TILs and CD103^+^ TILs and lymph node metastasis was almost the same as in the 98 cases including pT1a (Figure, Table). Risk factors for lymph node metastasis were similar to those in the 98 cases, including pT1a. Univariate analysis revealed that lymphatic invasion (+), budding grade (3), and CD8^+^ TILs (low) were risk factors for lymph node metastasis. Multivariate analysis confirmed that lymphatic invasion (+) and the number of CD8^+^ TILs (low) were independent risk factors for lymph node metastasis. (Supplementary Fig. 1a-d, Supplementary Table 1a, b).

Second, because of the strong impact of lymphatic invasion on the results, we examined the relationship between lymphatic invasion and tumor-infiltrating CD8^+^ T cells and found no predominant difference in lymphatic invasion and the number of CD8^+^ cells (*P* = 0.63). We examined whether the number of CD8^+^ cells is a risk factor for lymph node metastasis in cases without lymphatic invasion. Since there was only one case of lymph node metastasis in patients without lymphatic invasion, we did not find a statistically significant difference, but the results showed that CD8^+^ TILs are possibly a risk factor even in patients without lymphatic invasion (*P* = 0.08). (Supplementary Fig. 2 a, b).

## Discussion

In this study, we examined CD8^+^ TILs as a risk factor for lymph node metastasis in patients with SM-CRC. Our findings indicate low CD8^+^ TIL levels, as determined by the cutoff value from ROC curve analysis, to be a new, independent risk factor for lymph node metastasis. Furthermore, by combining the numbers of CD8^+^ and CD103^+^ TILs, we found a high PPV for lymph node metastasis in patients with low levels of both types of TILs and a high NPV in patients with high levels of both types of TILs. Thus, we most likely identified an effective subset of CD8^+^ T cells.

The few existing reports on the connection between TILs and lymph node metastasis have focused on breast cancer^16^ and melanoma^17^. In these reports, low TIL density was associated with lymph node metastasis. In breast cancer, CD8^+^ TILs have been found to have a negative correlation with lymph node metastasis^18^. Another study from 2020 reported a relationship between lymph node metastasis and TILs in early-stage breast cancer^16^. Conversely, TIL density and lymph node metastasis have been reported to have a low correlation in patients with esophageal squamous cell carcinoma^19^. One report has examined the relationship between TILs and lymph node metastasis in colorectal cancer; however, it examined CD3^+^ TILs and reported a low correlation^20^, likely because CD3^+^ cells include immunosuppressive T cells, such as Tregs^21^. Tregs are involved in the development and progression of cancer by inhibiting anticancer immunity^22^ and express CD3, CD4, CD25, and Foxp3, but not CD8, which is a cytotoxic T-cell marker^23^. The number of tumor-infiltrating CD8^+^ T cells is associated with tumor prognosis in many solid tumors^24^. Therefore, we thought that evaluating CD3^+^ T cells, including Tregs and cytotoxic T cells, may obscure the relationship between TILs and colorectal cancer lymph node metastasis, and evaluation of CD8 mostly expressed on cytotoxic T cells, alone does not lead to a major loss of direction. The results of a single cell analysis of immune cells from colorectal cancer (GSE188381) indicate that CD8 is almost expressed on cells expressing CD3. Single cell RNA-seq analysis of tumor infiltrating lymphocytes in colorectal cancer. The t-distributed stochastic neighbor embedding (t-SNE) plot showed that CD8A is almost expressed on CD3D, CD3E expressing cells (Supplementary Fig. 3). We hypothesized that evaluating only cytotoxic T cells would better clarify the relationship between lymph node metastasis and TILs. In this study, we did not examine pan-T cell markers such as CD3 and CD4 but only evaluated markers of cytotoxic T cells such as CD8 and CD103. We focused on CD8 cytotoxic T cells and found a relationship between CD8^+^ TILs and lymph node metastasis.

This study did not examine how CD8^+^ TILs or CD103^+^ TILs suppress lymph node metastasis, and this is one of the limitations of the present study.

Because tumors are heterogeneous, the site of assessment of the number of tumor-infiltrating T cells is important^25^. For example, previous reports have shown that the number of TILs in the tumor infiltrate is a more sensitive indicator of tumor metastasis than that in the center of the tumor^10^. Therefore, in this study, we evaluated the TIL count at the tumor invasion site.

We further evaluated a subset of cytotoxic T cells that express CD103. CD103^+^ CD8^+^ T cells are associated with an epithelial-derived carcinoma prognosis^11^ and CD103 alone contributes to prolonged survival in patients with bladder cancer^26^, ovarian cancer^27^, non-small cell lung cancer^28^, and melanoma^29^. Moreover, CD103 is expressed by the recognition of tumor antigens and is a marker of tumor-specific TILs^30^. However, in this study, CD103 alone was not a more accurate risk factor for lymph node metastasis than CD8. Additionally, this study featured only two cases of undifferentiated tumors, neither of which had lymph node metastasis. As CD103 was expected to predict lymph node metastasis to some extent, we would like to re-evaluate CD103 as a risk factor for lymph node metastasis by accumulating more cases.

We have a limitation of this study. The limitation is sample size was small. The present study was single-centered and included only recent cases from 2014 to 2019, after budding was included as a risk factor for lymph node metastasis in cancer with submucosal invasion, according to the guidelines of the Japanese Society for Cancer of the Colon and Rectum. We included the 98 cases of SM-CRC at our hospital for whom resected pathological specimens were available. Additionally, 11 cases (11.2%) of lymph node metastasis were observed, which is consistent with previously established rates of lymph node metastasis in SM-CRC of approximately 10%. However, the small number of cases with lymph node metastasis is a limitation of this study, and future studies with more cases are warranted.

In the present study, both the sensitivity and specificity of lymphatic invasion were high. The data set showed a strong relationship between lymphatic invasion and lymph node metastasis, as 10 of the 11 cases with lymph nodes had lymphatic invasions. In a previous report of 293 cases, the sensitivity was 0.6 (21/37) and the specificity 0.7 (188/256)^4^. This indicates that lymphatic invasion is significant in lymph node metastasis, but in the current data set, the relationship between lymphatic invasion and lymph node metastasis was particularly strong.

In conclusion, we examined CD8^+^ TIL levels as a risk factor for lymph node metastasis in patients with SM-CRC and showed that low CD8^+^ TIL levels are positively correlated with lymph node metastasis. The number of CD8^+^ TILs using a cutoff value identified by receiver operating characteristic curve analysis was an independent risk factor for lymph node metastasis.

## Material and methods

This study was approved by the Research Ethics Committee of Osaka University on October 9th, 2018 (approval no. 17448). The study was conducted in accordance with the principles of the 1964 Declaration of Helsinki and its subsequent amendments, or equivalent ethical standards. Written informed consent was obtained from all patients.

### Study design and patients

In this study, we retrospectively evaluated 180 patients with SM-CRC who underwent surgical resection between 2014 and 2019 at Osaka University Hospital.

### Immunohistochemical staining

Immunohistochemical staining was performed on formalin-fixed paraffin-embedded tissue Sects. (4.0 mm). After deparaffinization, antigen retrieval was performed using 10 mmol/L citrate buffer (pH 6), and intrinsic peroxidase activity was blocked using 3% H_2_O_2_ for 20 min, followed by nonspecific interaction blocking with a background sniper (Biocare Medical, Pacheco, CA, USA) for 10 min. Tissue sections were stained with anti-CD8 (ab75129, mouse, diluted 1:50) and CD103 (ab129202, rabbit, diluted 1:2,000) antibodies using the Vectastain ABC kit (Vector Laboratories, Burlingame, CA, USA). The number of CD8^+^ and CD103^+^ T cells in 10 HPFs at the tumor-invasive margins was counted using ImageJ software version 1.8.0 (NIH, Bethesda, MD, USA; http://imagej.nih.gov/ij). Specifically, we evaluated the image in the field of view with the highest number of CD8^+^ TILs in the most infiltrated area. We also used the cell count function in Image J to import the image and count the number of cells stained with DAB. The three most positive cell locations were identified in the 20 × field of view, and each was observed in the higher magnification field of view and measured with Image J. We measured the number of cells stained with DAB via Color Threshold with settings of Hue(200–250), Saturation(0–255), and Brightness(0–130). We counted the areas pointed out in red as shown in Supplementary Fig. 4.

### Statistical analysis

Statistically significant differences between categorical variables were determined using the χ^2^ test and between continuous variables using the Mann–Whitney test.

All statistical analyses were performed using R version 4.0.2 and JMPpro 14.0.0 (SAS Institute, Cary, NC, USA). Statistical significance was set at* P* < 0.05.

### Single cell RNA-seq analysis

The GSE108989 single-cell RNA seq data were download in Gene Expression Omnibus (https://www.ncbi.nlm.nih.gov/geo/).

The GSE108989 gene sets were analyzed using the R package Seurat version 3.1.5. The cited gene expression matrices from GSE108989 were read into R version 4.0.1 and converted to Seurat objects. To reduce dimensionality, principal component analysis was performed based on highly variable genes after scaling the data with respect to unique molecular identifier counts. Principle components were selected for downstream clustering based on the heatmap, jackstraw plot, and elbow plot of principal components in order to further reduce dimensionality by using the UMAP algorithm.

## Supplementary Information

Supplementary Information 1.

## Data Availability

All relevant data were included in this report.

## References

[1] Hashiguchi Y, Muro K, Saito Y (2020). Japanese Society for Cancer of the Colon and Rectum (JSCCR) guidelines 2019 for the treatment of colorectal cancer. Int. J. Clin. Oncol..

[2] Beaton C, Twine CP, Williams GL, Radcliffe AG (2013). Systematic review and meta-analysis of histopathological factors influencing the risk of lymph node metastasis in early colorectal cancer. Colorectal Dis..

[3] Ueno H, Hase K, Hashiguchi Y (2014). Novel risk factors for lymph node metastasis in early invasive colorectal cancer: A multi-institution pathology review. J. Gastroenterol..

[4] Fujino S, Miyoshi N, Ohue M (2017). A nomogram for predicting lymph node metastasis in submucosal colorectal cancer. Int. Surg..

[5] Jung CK, Jung SH, Yim SH (2016). Predictive microRNAs for lymph node metastasis in endoscopically resectable submucosal colorectal cancer. Oncotarget.

[6] Vesely MD, Kershaw MH, Schreiber RD, Smyth MJ (2011). Natural innate and adaptive immunity to cancer. Annu Rev Immunol..

[7] Hendry S, Salgado R, Gevaert T (2017). Assessing tumor infiltrating lymphocytes in solid tumors: A practical review for pathologists and proposal for a standardized method from the International immuno-Oncology Biomarkers Working Group: Part 2: TILs in melanoma, gastrointestinal tract carcinomas, non-small cell lung carcinoma and mesothelioma, endometrial and ovarian carcinomas, squamous cell carcinoma of the head and neck, genitourinary carcinomas, and primary brain tumors. Adv Anat Pathol..

[8] van der Leun AM, Thommen DS, Schumacher TN (2020). CD8^+^ T cell states in human cancer: Insights from single-cell analysis. Nat. Rev. Cancer..

[9] Barnes TA, Amir E (2017). HYPE or HOPE: The prognostic value of infiltrating immune cells in cancer. Br. J. Cancer.

[10] Sheu BC, Kuo WH, Chen RJ, Huang SC, Chang KJ, Chow SN (2008). Clinical significance of tumor-infiltrating lymphocytes in neoplastic progression and lymph node metastasis of human breast cancer. Breast.

[11] Takada K, Kashiwagi S, Asano Y (2020). Prediction of lymph node metastasis by tumor-infiltrating lymphocytes in T1 breast cancer. BMC Cancer.

[12] Galon J, Costes A, Sanchez-Cabo F (2006). Type, density, and location of immune cells within human colorectal tumors predict clinical outcome. Science.

[13] Amsen D, van Gisbergen KPJM, Hombrink P, van Lier RAW (2018). Tissue-resident memory T cells at the center of immunity to solid tumors. Nat Immunol..

[14] Gorfu G, Rivera-Nieves J, Ley K (2009). Role of β7 integrins in intestinal lymphocyte homing and retention. Curr. Mol. Med..

[15] Jou J, Diehl AM (2010). Epithelial-mesenchymal transitions and hepatocarcinogenesis. J. Clin. Invest..

[16] Wong SHM, Fang CM, Chuah LH, Leong CO, Ngai SC (2018). E-cadherin: Its dysregulation in carcinogenesis and clinical implications. Crit. Rev. Oncol. Hematol..

[17] Jie D, Zhongmin Z, Guoqing L (2013). Positive expression of LSD1 and negative expression of E-cadherin correlate with metastasis and poor prognosis of colon cancer. Dig. Dis. Sci..

[18] Taylor RC, Patel A, Panageas KS, Busam KJ, Brady MS (2007). Tumor-infiltrating lymphocytes predict sentinel lymph node positivity in patients with cutaneous melanoma. J. Clin. Oncol..

[19] Min BH, Yang JW, Min YW (2020). Nomogram for prediction of lymph node metastasis in patients with superficial esophageal squamous cell carcinoma. J. Gastroenterol. Hepatol..

[20] Di Caro G, Bergomas F, Grizzi F (2014). Occurrence of tertiary lymphoid tissue is associated with T-cell infiltration and predicts better prognosis in early-stage colorectal cancers. Clin. Cancer Res..

[21] Deng L, Zhang H, Luan Y (2010). Accumulation of foxp3^+^ T regulatory cells in draining lymph nodes correlates with disease progression and immune suppression in colorectal cancer patients. Clin. Cancer Res..

[22] Sakaguchi S (2000). Regulatory T cells: Key controllers of immunologic self-tolerance. Cell.

[23] Ohue Y, Nishikawa H (2019). Regulatory T (Treg) cells in cancer: Can Treg cells be a new therapeutic target?. Cancer Sci..

[24] Fridman WH, Pagès F, Sautès-Fridman C, Galon J (2012). The immune contexture in human tumours: Impact on clinical outcome. Nat. Rev. Cancer..

[25] Chen Z, Zhang C, Pan Y (2016). T cell receptor β-chain repertoire analysis reveals intratumour heterogeneity of tumour-infiltrating lymphocytes in oesophageal squamous cell carcinoma. J Pathol..

[26] Wang B, Wu S, Zeng H (2015). CD103^+^ tumor infiltrating lymphocytes predict a favorable prognosis in urothelial cell carcinoma of the bladder. J. Urol..

[27] Webb JR, Milne K, Watson P, Deleeuw RJ, Nelson BH (2014). Tumor-infiltrating lymphocytes expressing the tissue resident memory marker CD103 are associated with increased survival in high-grade serous ovarian cancer. Clin. Cancer Res..

[28] Djenidi F, Adam J, Goubar A (2015). CD8^+^ CD103^+^ tumor–infiltrating lymphocytes are tumor-specific tissue-resident memory T cells and a prognostic factor for survival in lung cancer patients. J. Immunol..

[29] Edwards J, Wilmott JS, Madore J (2018). CD103^+^ tumor-resident CD8^+^ T cells are associated with improved survival in immunotherapy-naïve melanoma patients and expand significantly during anti–pd-1 treatment. Clin. Cancer Res..

[30] Ling KL, Dulphy N, Bahl P (2007). Modulation of CD103 expression on human colon carcinoma-specific CTL. J. Immunol..

