# Supplementary material for: Tumor-infiltrating T cells as a risk factor for lymph node metastasis in patients with submucosal colorectal cancer
Source: Sci Rep. 2023 Feb 6;13:2077. doi: 10.1038/s41598-023-29260-1 (PMC9902519; doi:10.1038/s41598-023-29260-1)
Supplement: Supplementary file 1 — Supplementary Information 1. [file 41598_2023_29260_MOESM1_ESM.docx]

Supplementary Fig.1 The relationship between CD8^+^ and CD103^+^ TIL and lymph node metastasis in pT1b 78 cases. (a) The number of CD8^+^ TILs and the number of lymph node metastases. (b) The number of CD8^+^ TILs and presence of lymph node metastasis (p = 0.02) (c) The number of CD103^+^ TILs and the number of lymph node metastases. (d) The number of CD103^+^ TILs and presence of lymph node metastasis (p = 0.04)

a.


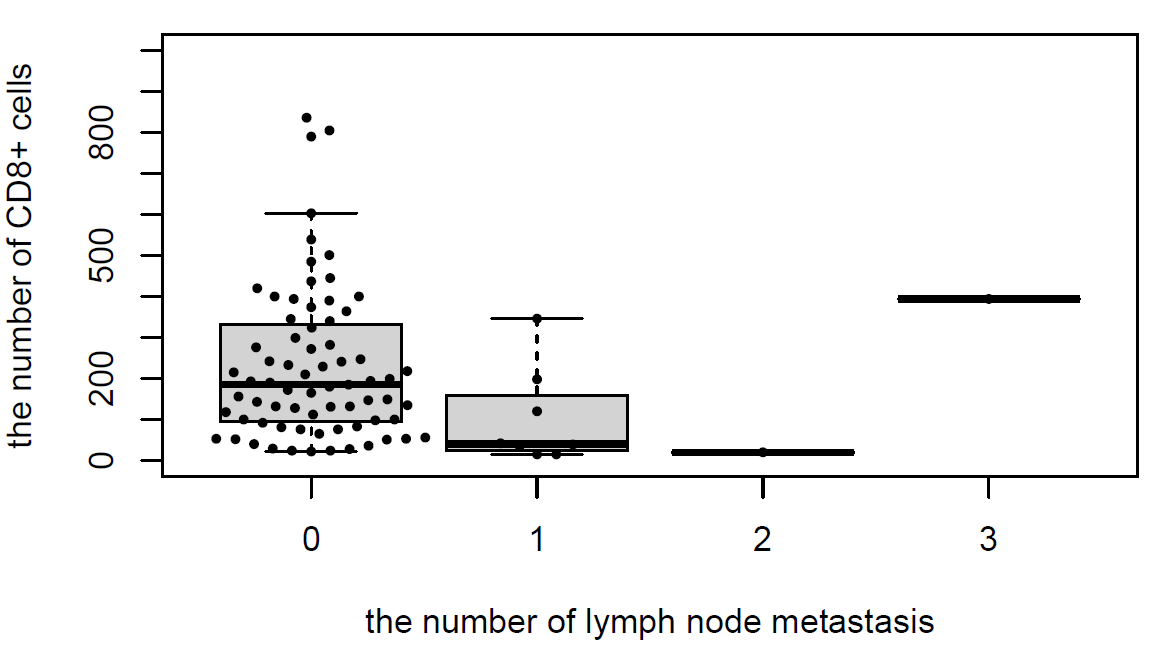


b.


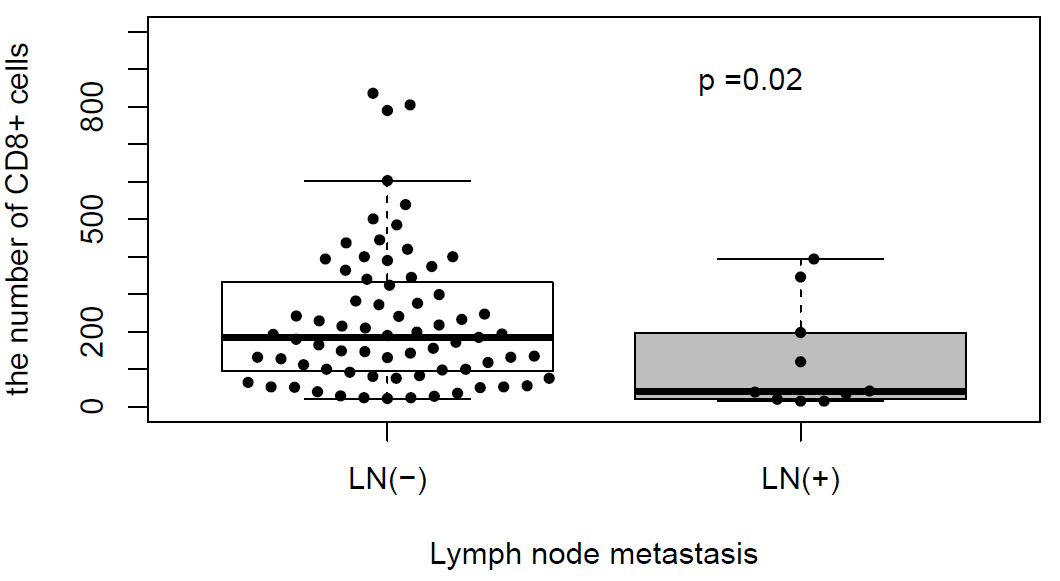


c.


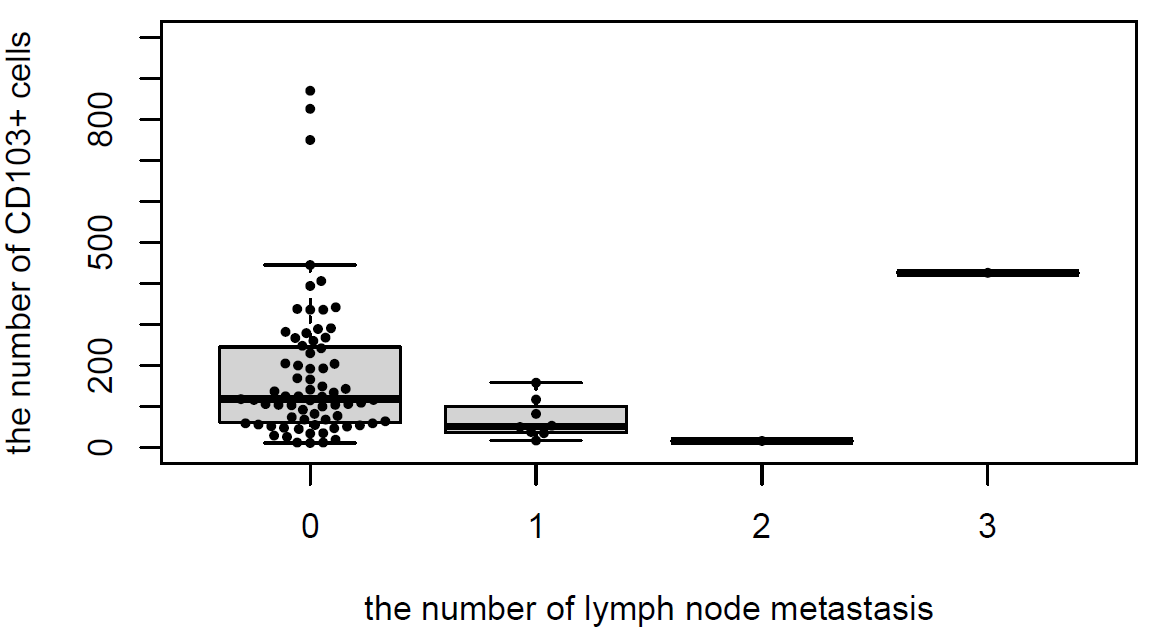


d.


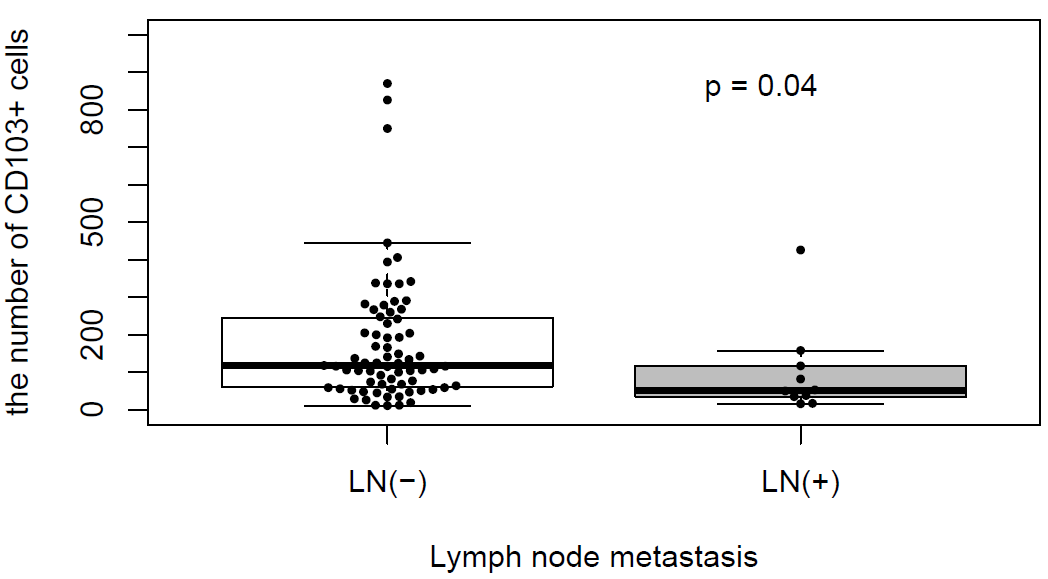


Supplementary Fig.2 (a) The relationship between the number of CD8^+^ TIL and lymphatic invasion. (b) The number of CD8^+^ TILs and lymph node metastasis in cases without lymphatic invasion.

a.


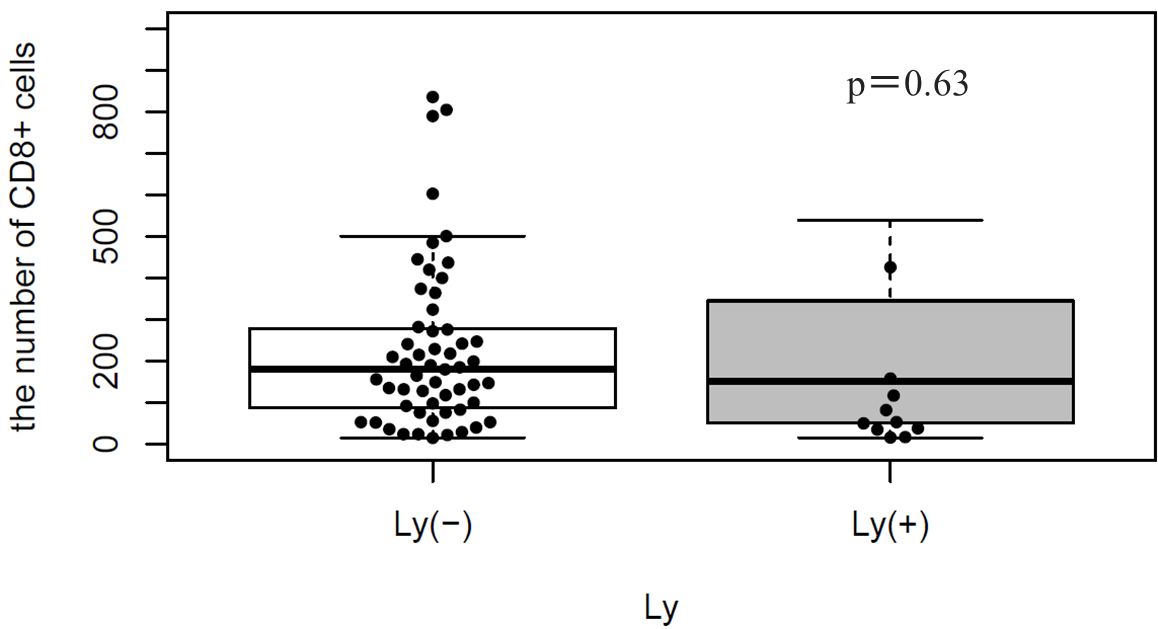


b.


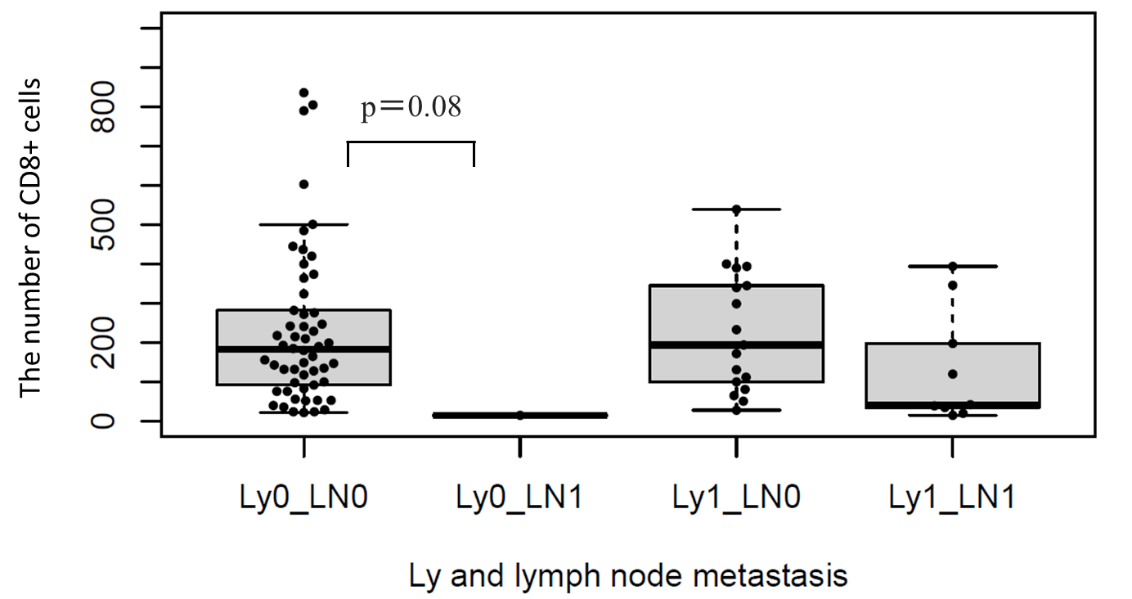


Supplementary Fig.3

Analysis of GSE108989 single cell RNA-seq data (Single T-cell Analysis in 12 colorectal cancers) revealed that gene expression of CD8A (CD8) and ITGAE (CD103) is strongly associated with cytotoxic cytokines such as GZMB and PFR1.


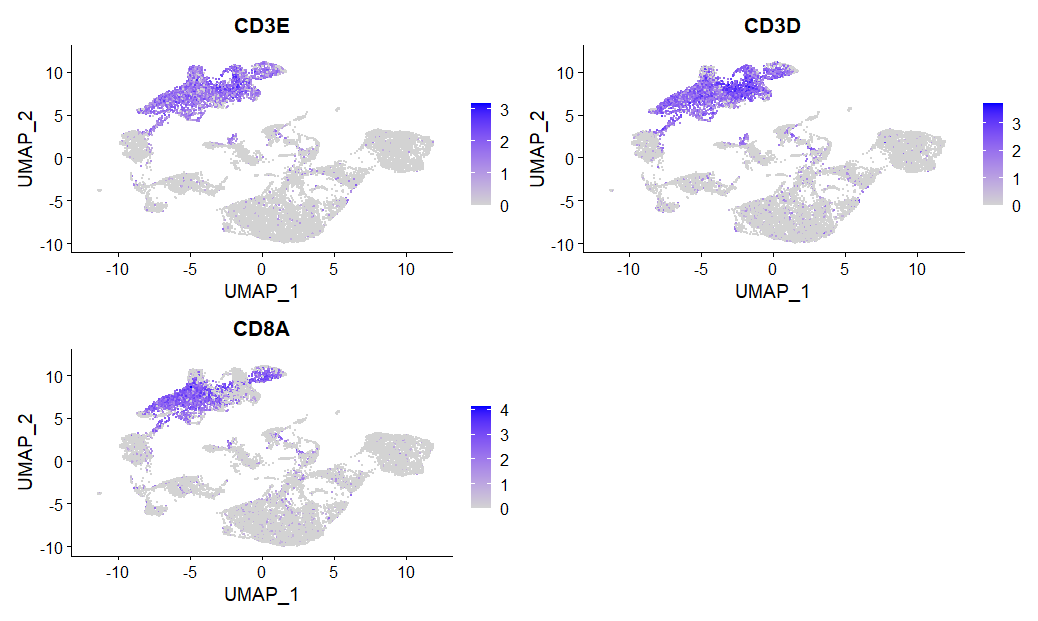


Supplementary Fig.4

Example of a count of DAB-positive cells by immunohistochemical staining


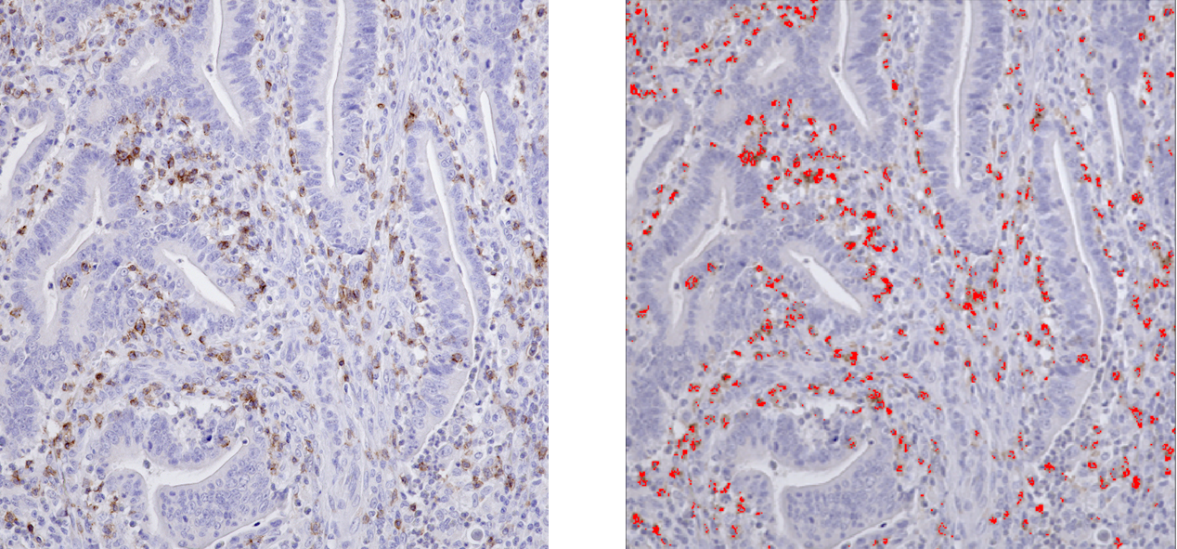


Supplementary Table 1a Variables of lymph node metastasis in pT1b cases (univariate and multivariate analyses)

|  | **Univariate** | | |  | | **Multivariate** | | | | | |  |
| --- | --- | --- | --- | --- | --- | --- | --- | --- | --- | --- | --- | --- |
| **Variable** | **OR** | **95% CI** | **p-value** |  | **OR** | | **95% CI** | **p-value** | **OR** | **95% CI** | **p-value** | |
| Age (≧65 years) | 1.63 | 0.43–6.85 | 0.476 |  |  | |  |  |  |  |  | |
| Sex (female) | 0.86 | 0.21–3.28 | 0.827 |  |  | |  |  |  |  |  | |
| Location (rectum) | 0.70 | 0.14–2.75 | 0.624 |  |  | |  |  |  |  |  | |
| Histological type (por, muc) | - | - | - |  |  | |  |  |  |  |  | |
| Histological type (tub2, por, muc) | 0.73 | 0.17 - 2.76 | 0.320 |  |  | |  |  |  |  |  | |
| Greatest diameter (≧20 mm) | 0.39 | 0.06 - 1.77 | 0.27 |  |  | |  |  |  |  |  | |
| Depth of invasion (≧3000μm) | 1.37 | 0.35 - 5.32 | 0.64 |  |  | |  |  |  |  |  | |
| Lymphatic invasion (+) | 28.6 | 4.87 - 546 | <0.01 |  | 47.2 | | 5.08 - 1630 | 0.01 | 38.4 | 5.25 - 905 | <0.01 | |
| Vascular invasion (+) | 1.02 | 0.14 - 4.64 | 0.98 |  |  | |  |  |  |  |  | |
| Budding grade (2, 3) | 1.62 | 0.32 - 6.75 | 0.53 |  |  | |  |  |  |  |  | |
| Budding grade (3) | 15.25 | 1.32 – 351 | 0.03 |  | 35.2 | | 0.68 - 4882 | 0.12 | 23.9 | 0.66 - 2259 | 0.13 | |
| Preoperative CEA (≧2 ng/ml) | 0.8 | 0.1 - 16.7 | 0.85 |  |  | |  |  |  |  |  | |
| Preoperative CA19-9 (≧10 ng/ml) | 0.77 | 0.09 - 16.6 | 0.83 |  |  | |  |  |  |  |  | |
| The number of CD8⁺ TILs (<46/10 HPF) | 13.7 | 3.2 - 66.4 | 0.001 |  | 31.2 | | 3.65 - 763 | 0.01 |  |  |  | |
| The number of CD103⁺ TILs (<46/10 HPF) | 4.46 | 1.1 - 18.4 | 0.03 |  |  | |  |  | 7.78 | 1.2 - 71.4 | 0.04 | |

OR, odds ratio; CI, confidence interval; CEA, carcinoembryonic antigen; CA19-9, Carbohydrate Antigen 19-9; CD, cluster of differentiation; TIL, tumor-infiltrating lymphocytes; HPF, high-power fields

Supplementary Table 1b Sensitivity, specificity, positive predictive value (PPV), and negative predictive value (NPV) of risk factors

|  | Sensitivity | Specificity | PPV | NPV |
| --- | --- | --- | --- | --- |
| Histological type (por, muc) | 0.0 | 97.2 | 0.0 | 87.3 |
| Histological type (tub2, por, muc) | 40.0 | 52.1 | 10.5 | 86.0 |
| Depth of invasion (≧3000 μm) | 50.0 | 57.7 | 14.3 | 89.1 |
| Lymphatic invasion (+) | 90.0 | 76.1 | 34.6 | 98.2 |
| Vascular invasion (+) | 20.0 | 80.3 | 12.5 | 87.7 |
| Budding grade (2, 3) | 30.0 | 69.0 | 18.8 | 87.5 |
| Budding grade (3) | 20.0 | 85.9 | 66.7 | 88.4 |
| CD8 | 60.0 | 90.1 | 46.2 | 94.1 |
| CD103 | 50.0 | 81.7 | 27.8 | 92.1 |
| CD8 and CD103 (LL/HH) | 40.0 | 97.2 | 66.7 | 92.0 |
| CD8 and CD103 (Other/HH) | 70.0 | 74.6 | 28.0 | 94.6 |

CD, cluster of differentiation; LL, numbers of both CD103^+^ and CD8^+^ TILs were low; HH, numbers of both CD8^+^ and CD103^+^ TILs were high
